# Education: The Key of SBCCV. Performance of BJCVS, The Facts

**DOI:** 10.21470/1678-9741-2017-0502

**Published:** 2017

**Authors:** Domingo M. Braile

**Affiliations:** 1 Editor-in-Chief - BJCVS

In April 2017, the 44^th^ Congress of the Sociedade Brasileira de Cirurgia Cardiovascular (SBCCV) took place in the amazing city of Rio de Janeiro, demonstrating once more the success of this event already consolidated in the Brazilian Cardiovascular Surgery community.

Among the traditional activities, we had the Scientific Methodology Course this year, focused primarily for the candidates approved in the first phase of the test to obtain the Specialist Degree in Cardiovascular Surgery.

It was not a surprise to see many Cardiovascular Surgeons also interested in the subject matter, present in the classroom.

The idea of teaching this course arose from the lack of specific knowledge of our associates when writing their manuscripts to fulfill the exigencies to obtain the Specialist Title. An orientation in basic notions of research, statistics and data analysis seemed to be necessary, giving the authors greater confidence when writing their manuscripts.

To our great satisfaction, we can realize that in each edition, a greater adherence and awareness of the importance in master basic rules for quality publications by our authors.

This is one of the objectives of the BJCVS, as well as educate, teach and encourage researches in our community.

Taking advantage of the Congress, we also had the annual meeting of the Editorial Board of the BJCVS, held on April 21, 2017. Several issues were discussed, following the main motto of the Board: the participation of the BJCVS young team, showing the results achieved in one year of work, which certifies we are on the right track, investing in the youth, sharing actions, and exchanging experiences with the new, with the future.

Today, the BJCVS has a team of young collaborators, from the Junior Editor to the team that manages the Blog and our social networks.

They could increase the accesses of BJCVS in a short period of time, sharing texts of excellent quality, with varied current subjects, promoting reading and knowledge.

The BJCVS, along with the SBCCV, will continue to invest and encourage the education of all associates, academics, residents and surgeons, showing that Cardiovascular Surgery has a consolidated present and future due to the professionals' quality who perform it.

Besides the success achieved by this event, activity that shows the vitality of our Society, we also want to refer to the evolution of the BJCVS as the vehicle that every two months shows the scientific production of our associates, and from the national and international authors, more and more frequent in our pages.

The BJCVS improves in every edition, a reason of great pride for our entire community, showing that we can surpass ourselves even while experiencing periods of profound political, economic and social crisis in our country.

Growing during crises is a task for personalities endowed with determination and character, capable of believing in the transforming capability we own.

It is encouraging to see that even in this atmosphere, we overcome ourselves, as demonstrated by the insertion of the BJCVS in the international scope.

To publish more quality work and to increase the citation of BJCVS articles, we have sent to all those who submit manuscripts to our journal, whether they are accepted articles or not, a message encouraging them to send other articles, besides emphatically asking them to cite the articles of the BJCVS in all their publications.

We have had very positive results, as we can see, among others, in the correspondence of an author from Uruguay, reproduced below.

"Dear Prof. Domingo, Braile

I appreciate your letter very much. We will re-write the manuscript trying to fulfill all the reviewer comments.

We are a very active group who have constant and frequent publications. Due to your letter and with the intention of strengthening the BJCVS I commit myself to increase the number of manuscripts to your journal.

We consider the Brazilian Journal of Cardiovascular Surgery an ideal journal to distribute results from South American regarding Cardiovascular Surgery.

Sincerely yours,

Victor Dayan".

We present below the BJCVS performance from January 1 to May 31, 2017, demonstrating how, step by step, we are becoming the leading publication of our specialty, throughout the Southern Hemisphere.

Total articles received: 162 of 14 countries.

**Fig. 1:**
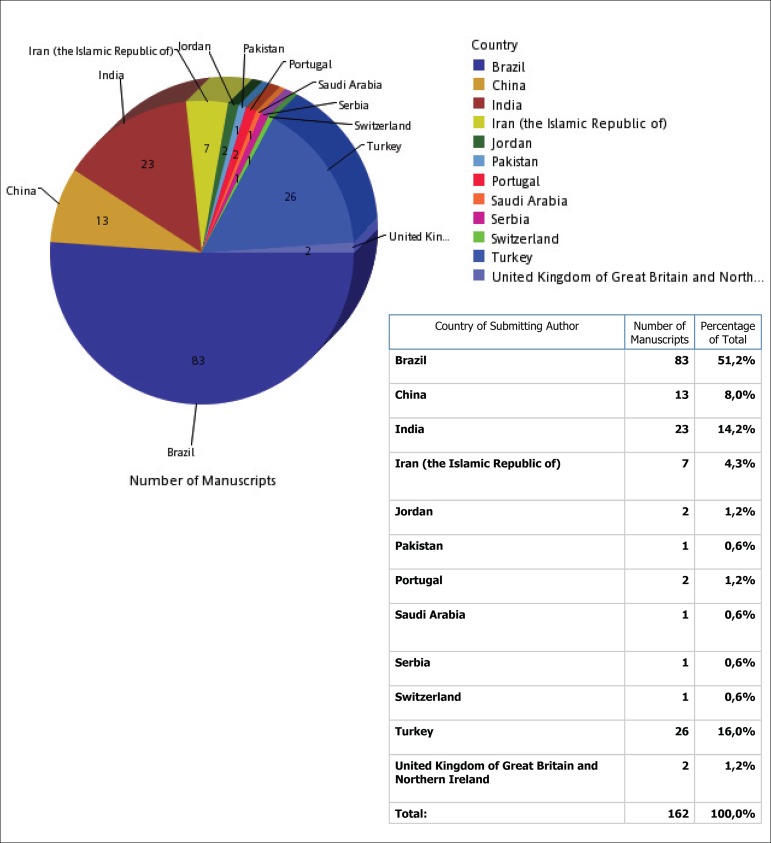
Countries that submitted manuscripts to the BJCVS between January 1 and May 31.

Countries from which we received articles: Brazil; China; India; Iran; Jordan; Pakistan; Portugal; Saudi Arabia; Serbia; Switzerland; Turkey; United Kingdom of Great Britain; Northern Ireland (Figure 1).

**Fig. 2:**
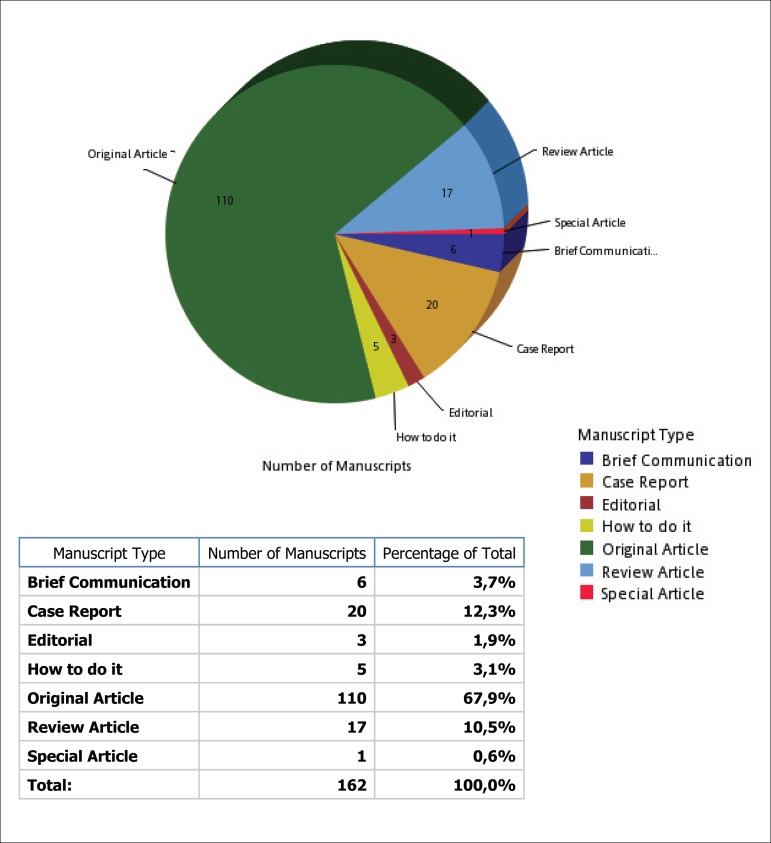
Manuscripts received by category between January 1 and May 31.

Regarding the categories of manuscripts, we also had, among others: 110 original articles, 20 case reports and 17 review articles (Figure 2).

Showing the rigor with which Reviewers, Associate Editors and Editor-in-Chief have been conducting the evaluation of the articles, among the 162 manuscripts received, only 30 were accepted, with an 80% rejection rate!

Twelve were accepted directly at this time because they proceed from the former GN1 platform, where they were exhaustively evaluated by our reviewers and Associate Editors.

**Fig. 3:**
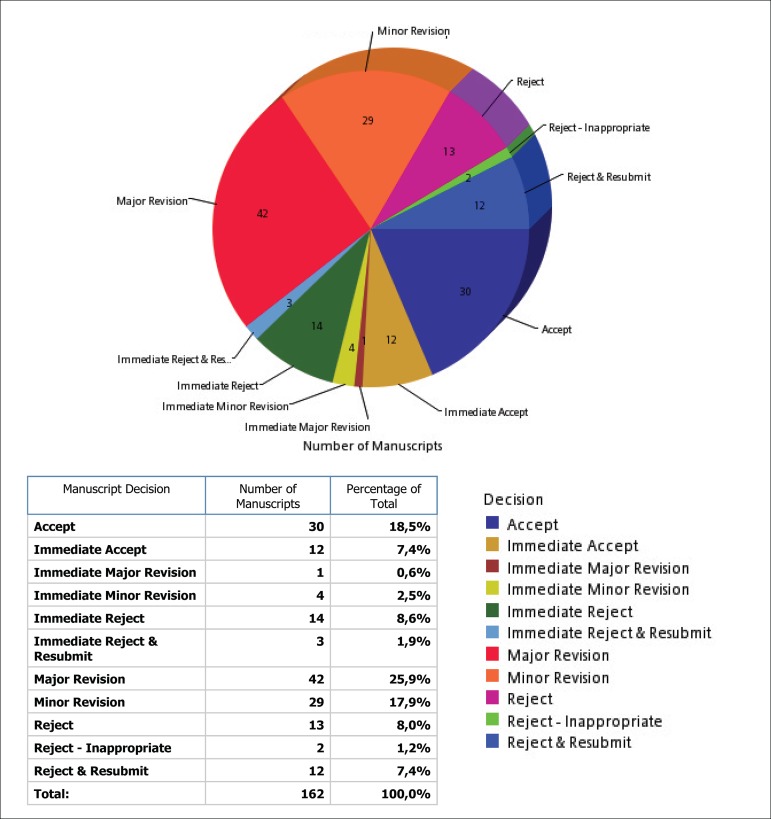
Manuscripts accepted or rejected between January 1 to May 30.

The facts that contributed to this high rejection rate were the low quality of the works, and the almost total rejection of the Case Reports, which add few knowledge, and are harmful to the Impact Factor (Figure 3).

We can say with certainty that we are on the right track!

I would like to commend the tireless dedication of the Associate Editors, Reviewers, and the support team who spare no efforts, promoting the BJCVS to occupy the prominent place it deserves. But this would be impossible without the unrestricted support of the SBCCV Directory, and Associates, who always did their best to help us.

Have a great reading.

Kind regards,

**Domingo M. Braile** ^1^Editor-in-Chief - BJCVS

